# Small-Molecule Inhibitors of the Type III Secretion System

**DOI:** 10.3390/molecules200917659

**Published:** 2015-09-23

**Authors:** Lingling Gu, Shanshan Zhou, Lanping Zhu, Cuirong Liang, Xin Chen

**Affiliations:** School of Pharmaceutical Engineering and Life Science, Changzhou University, Changzhou 213164, China; E-Mails: kikyo1210@sina.com (L.G.); sunnychou33@sina.com (S.Z.); zhulanping91@163.com (L.Z.); Liang_cuirong@163.com (C.L.)

**Keywords:** type III secretion system, pathogens, small-molecule inhibitors, effector proteins, Gram-negative bacteria, virulence, antibacterial agents

## Abstract

Drug-resistant pathogens have presented increasing challenges to the discovery and development of new antibacterial agents. The type III secretion system (T3SS), existing in bacterial chromosomes or plasmids, is one of the most complicated protein secretion systems. T3SSs of animal and plant pathogens possess many highly conserved main structural components comprised of about 20 proteins. Many Gram-negative bacteria carry T3SS as a major virulence determinant, and using the T3SS, the bacteria secrete and inject effector proteins into target host cells, triggering disease symptoms. Therefore, T3SS has emerged as an attractive target for antimicrobial therapeutics. In recent years, many T3SS-targeting small-molecule inhibitors have been discovered; these inhibitors prevent the bacteria from injecting effector proteins and from causing pathophysiology in host cells. Targeting the virulence of Gram-negative pathogens, rather than their survival, is an innovative and promising approach that may greatly reduce selection pressures on pathogens to develop drug-resistant mutations. This article summarizes recent progress in the search for promising small-molecule T3SS inhibitors that target the secretion and translocation of bacterial effector proteins.

## 1. Introduction

Although antibiotic therapy is the most commonly-used strategy to control infectious pathogens, most antibiotics affect cellular processes of microorganisms and, therefore, kill them, which becomes a strong selective pressure to develop resistance against antibiotics [1]. In order to contain the risk of increasing antibiotic resistance, targeting bacterial virulence factors instead of bacterial survivability provides a novel alternative approach for the development of new antimicrobials, because virulence-specific therapeutics create less selection pressure for antibiotic-resistant mutations [2,3]. The discovery of bacterial secretion systems is an important milestone in studies on the mechanisms of bacterial pathogenesis. Most enterobacterial pathogens, such as animal pathogens *Pseudomonas aeruginosa*, *Salmonella Typhimurium* and *Yersinia pestis*, and plant pathogens *Dickeya dadantii*, *Erwinia amylovora* and *Pseudomonas syringae*, possess at least one type III secretion system (T3SS) as a major virulence determinant [4,5]. Through this protein secretion/injection system, pathogens translocate their virulence factors (effectors) directly into host cells, enabling infection by subverting cells’ defense mechanisms [6,7]. T3SS represents a particularly appealing target for antimicrobial agents, because the antimicrobial therapies using T3SS-specific inhibitors would affect the virulence rather than the viability of pathogens, creating low selective pressure for developing drug resistance [2,3].

## 2. The Components of T3SS

**Figure 1:**
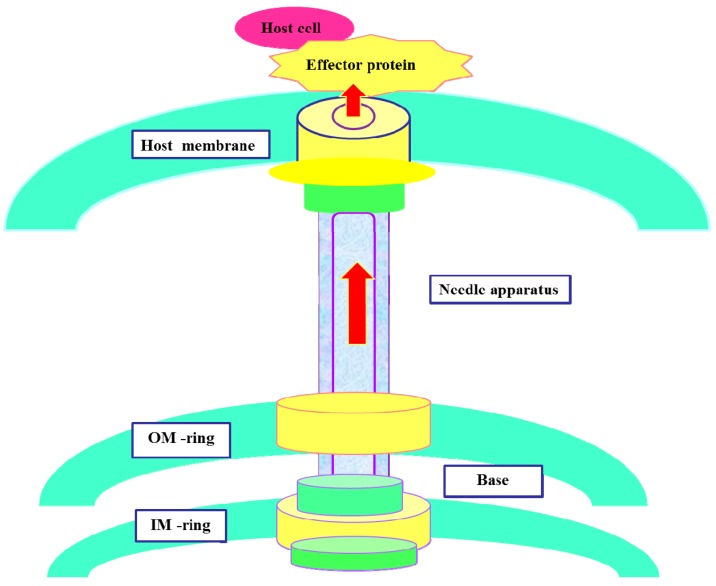
Schematic diagram of the T3SS in which the needle apparatus is contacting the host cell. OM, outer membrane; IM, inner membrane.

As one of the most complicated currently known protein secretion systems, the T3SS is usually encoded by a 30–40-kbp gene and exists in the bacterial chromosome or plasmid in the form of a pathogenicity island [8]. T3SSs of animal or plant pathogens possess many highly conserved main structural components, which are comprised of more than 20 proteins. Various pathogenic T3SSs are very similar in their structures, and their syringe-like appearance in transmission electron microscope images has led them to be called needle complexes (NCs) [9]. The core of the T3SS injection device is a needle-shaped complex that consists of a multi-ring base and a needle protrusion. The two parts are connected by an external needle, which protrudes from the bacterial surface [10,11,12] (Figure 1). The multi-ring base is composed of a pair of inner and outer rings that span the bacterial inner membrane (IM) and outer membrane (OM), and the two rings are connected by a rod-shaped structure passing through the IM and OM [13,14]. Needle protrusions have a straight hollow cylindrical structure containing the narrow center channel (~28 Å in diameter), specialized in transporting secretory proteins. The center channel stretches from the bottom of the ring structure to the top of the needle tip [15].

Although the three-dimensional structure of the T3SS needle is currently unknown, due to its inherent non-crystallinity (for X-ray crystallography) and insolubility (for solution NMR analysis), a higher-resolution atomic model of the *Salmonella* needle has been reported by American and German researchers [16]. They used solid-state NMR, electron microscopy and Rosetta model techniques to reveal the key features of the *Salmonella* needle: (1) the needle is composed of PrgI protomers with an α-helical hairpin head structure; (2) the needle has an 80 Å outer diameter with a 25 Å lumen; (3) the 80 residue subunits form a right-handed helical assembly with roughly 11 subunits per two turn; and (4) the *N*-terminus of PrgI is positioned on the surface of the needle, while the highly conserved *C*-terminus points towards the interior [16].

A key component of all T3SSs is the translocon, a proteinaceous channel (20–30 Å) that is inserted into the target membrane and that allows the passage of effectors into the host cell [12]. Another essential component of T3SS is a family of customized chaperones that specifically bind a ~100-amino acid domain at the N-terminus of the cognate secretion substrates [17]. Chaperones belong to three different classes: Class I chaperones recognize effectors; class II chaperones interact with translocators; and class III chaperones sequester the needle-forming proteins [18]. The chaperones of the translocon proteins are class II chaperones, and they are responsible for partitioning the intracellular pools of the translocon proteins in order to prevent their premature degradation [12]. Effector proteins travel in an unfolded or partially-folded conformation through the type III secretion channels, and the chaperones may keep the proteins in a secretion-competent conformation, probably by preventing them from folding. For example, Feldman *et al*. have confirmed that Yop effectors are never folded on their way to the *Y. enterocolitica* secretion system. Instead, SycE (the cognate chaperone of YopE) binds to the proteins and keeps the proteins in a partially-folded or unfolded conformation [19].

## 3. Action Mechanism of T3SS

**Figure 2:**
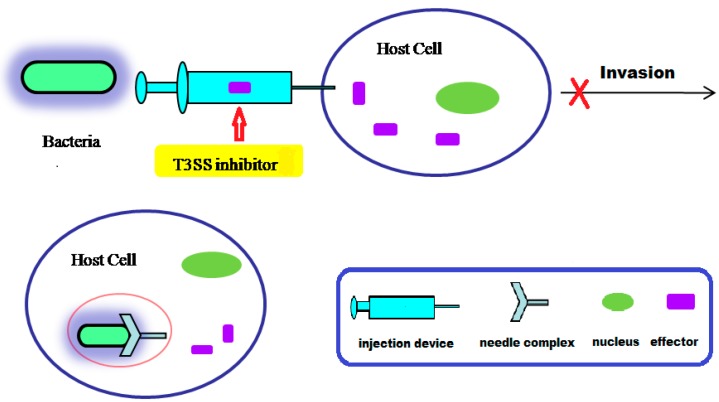
Schematic diagram of anti-virulence strategies by using T3SS inhibitors in Gram-negative bacterial pathogens.

Highly conserved T3SS is a secretion system consisting of a variety of components, and the secretion follows a single-step, sec-independent model [18]. The secretory signal of T3SS secretory protein is not dependent on the signal peptide, but occurs through a 15–20-amino acid domain at the *N*-terminus of the secretory protein. The secreted proteins are not cleaved in the cytoplasm, but are transported to the cell surface from the cytoplasm [15]. The T3SS injection device forms a temporary structure when bacteria interact with the host cell. The injection device is activated for assembly under physiological conditions [15]; However, under low calcium conditions, the injection device can also be activated. The needle complex of T3SS secretes and translocates protein effectors into the host cytoplasm, interfering with the function of the host cell (Figure 1) [20,21,22,23]. Hundreds of effector proteins have been identified as potential T3SS substrates from diverse bacterial pathogens. Pathogenic T3SS effector proteins can alter signal transduction pathways in order to evade the host defense system. Some bacterial effectors can enter into host cells and destroy their immune system, ultimately inducing host cell death (Figure 2) [24]. Although the detailed molecular mechanisms of T3SS are still unclear, current research has shown that effector translocation by T3SS is a key way that Gram-negative bacteria can infect host cells [23,24]. T3SS has emerged as an attractive target for developing novel anti-virulence agents. In terms of therapeutic strategies, T3SS inhibitors might be used to prevent effector proteins from invading the host cell through active or passive immunization, so that the pathogens lose the ability to infect host cells (Figure 2).

In 2014, American pharmaceutical company Kalobios reported antibody KB001-A as a *P. aeruginosa* T3SS inhibitor, and it is currently in phase II clinical trials for treating patients who have respiratory tract inflammation and cystic fibrosis (CF) caused by chronic *P. aeruginosa* infection [25]. To the best of our knowledge, this is the first T3SS inhibitor drug used in humans, suggesting an alternative to traditional antibiotics for treating bacterial infection. The action mechanism of T3SS inhibitors is very different from that of conventional antibiotics, because these inhibitors specifically target the virulence of bacteria, rather than their viability, making selective pressure on bacteria low and reducing the likelihood of bacterial resistance. T3SS inhibitors, especially small-molecule inhibitors, have attracted much attention in recent years, and this review summarizes several classes of small-molecule T3SS inhibitors reported in the literature between 2003 and 2015. 

## 4. Small Molecule Inhibitors of T3SS

### 4.1. Salicylidene Acyl Hydrazides as T3SS Inhibitors

**Figure 3:**
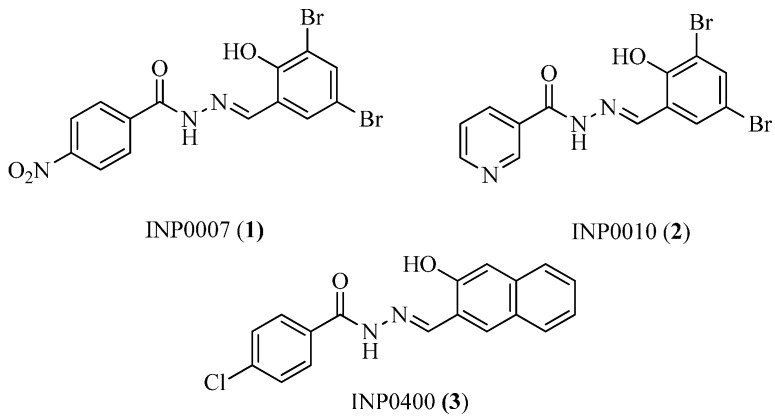
Structures of INP0007 (**1**), INP0010 (**2**) and INP0400 (**3**).

Salicylidene acyl hydrazides were reported as the first class of small-molecule T3SS inhibitors that can block one or more effector proteins. Using a luciferase reporter gene assay [26] in viable *Yersinia pseudotuberculosis*, Kauppi *et al.* [27] screened a chemical library of 9400 compounds and identified several salicylidene acyl hydrazide derivatives, such as INP0007 (**1**) and INP0010 (**2**) (Figure 3). These derivatives dramatically suppressed the reporter gene signal expressed by the *yopE* promoter, as well as effector protein secretion at low micromolar concentrations without affecting bacterial growth. INP0007 (**1**) blocked *Yersinia* T3SS secretion and prevented bacteria from invading HeLa cells. When the uninfected HeLa cells were incubated in the presence of INP0007 (50 μM), INP0007 strongly reduced the YopE-induced cytotoxic reaction in the cells [28]. This result implies that INP0007 can curb the translocation of several Yop effectors proteins, such as YopE. INP0010 (**2**) blocked microinjection of Yops into the cytosol of the target cell and inhibited replication of *Chlamydia* in Hep-2 cells [29].

Another salicylidene acyl hydrazide, INP0400 (**3**) (Figure 3), inhibited the intracellular replication and infectivity of *Chlamydia trachomatis*. Interestingly, the inhibitor has different effects at different stages of the infectious cycle of *C. trachomatis* [30]: when given at the time of infection, INP0400 partially blocked the entry of elementary bodies (EBs) into McCoy cells (host cells). In the mid-cycle, the inhibitor suppressed secretion of the effector IncA and homotypic vesicular fusions mediated by this protein. In the late phase, treatment resulted in detachment of reticulate bodies (RBs) from the inclusion membrane, ultimately preventing the conversion of RB to EB, thereby significantly reducing the infectivity of *C*. *trachomatis.*

More Gram-negative pathogens have been investigated using salicylidene acyl hydrazide inhibitors [31,32]. Hudson *et al.* found that INP0007 and INP0400 inhibited T3SS-mediated secretion of proteins without affecting the growth of bacteria in *Salmonella* [32]. Subsequently, they confirmed that the two inhibitors were capable of inhibiting secreted proteins via T3SS-mediated and inflammatory response by *Salmonella in vitro*. These results have demonstrated that salicylidene acyl hydrazides have the capacity to inhibit T3SSs in multiple species of pathogens and could serve as promising starting points for drug development [29].

### 4.2. N-Hydroxybenzimidazoles as T3SS Inhibitors

LcrF is a member of the AraC family of transcription activators [33] and is a multiple adaptational response (MAR) transcription factor associated with virulence in *Yersinia pestis* and *Y. pseudotuberculosis* [34]. Upon contacting host cells or changing temperature, LcrF is expressed and activates the expression of *Yersinia* cytotoxic Yop proteins, such as YopH, YopJ and YopE [35,36]. Yops are secreted into host cells via T3SS and cause cellular apoptosis [37,38]. *Y. pestis* is the causative agent of plague and raises serious concerns regarding its potential use as a biological weapon by terrorists [39].

**Figure 4:**
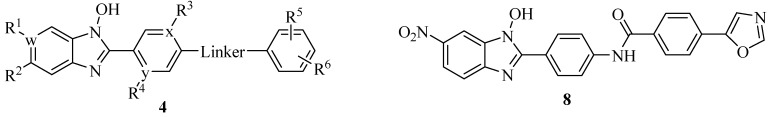
Structure of *N*-hydroxybenzimidazoles.

Kim *et al.* synthesized a series of *N*-hydroxybenzotriazole derivatives [40] (general structure shown as **4** in Figure 4) and identified some derivatives as potent LcrF inhibitors using a primary cell-free LcrF-DNA binding assay, as well as a T3SS-dependent whole cell assay (T3SS-dependent *Y. pseudotuberculosis* cytotoxicity assay) [34]. Lead Compounds **5**, **6** and **7** exhibited very similar inhibitory activity against both LcrF and ExsA (Table 1); ExsA is an MAR transcription factor found in *P. aeruginosa* and has 85% identity and 92% similarity with LcrF. However, these compounds have very weak inhibitory activity against SlyA, a member of the MarR family of transcription factors in *Salmonella* spp. The DNA binding motif of SlyA is different from that of MAR proteins [41,42]. These results indicated that the *N*-hydroxybenzimidazole inhibitors are non-DNA binding and specifically target MAR proteins. In a murine model of *Y. pseudotuberculosis* pneumonia, Compound **5** and another *N*-hydroxybenzimidazole derivative, **8** (Figure 4), significantly reduced the bacterial burden in the lungs, offering a dramatic survival advantage [43]. The inhibitors attenuated the T3SS-mediated virulence without affecting the normal bacterial growth in *Y. pseudotuberculosis* and did not demonstrate toxicity against mammalian cells [43]. Since the members of the MAR family of transcription factors play central roles in pathogenesis across bacterial genera, the *N*-hydroxybenzimidazole inhibitors could have broad applicability as, for example, lead compounds for preclinical studies of preventing *Y.* spp. infection.

### 4.3. Phenoxyacetamides as T3SS Inhibitors 

**Figure 5:**
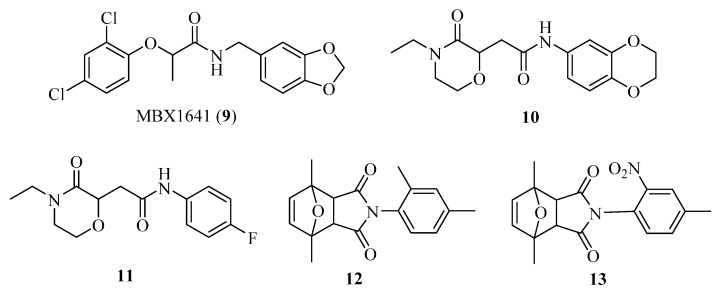
Structures of **9**–**13**.

The opportunistic pathogen *P. aeruginosa* is the leading cause of hospital-acquired infections by Gram-negative bacteria, and T3SS is the major virulence factor contributing to infections [44]. There are four T3SS effectors in *P. aeruginosa* strains: ExoS, ExoT, ExoY and ExoU. ExoU and ExoS contributed significantly to persistence, dissemination and mortality, and ExoT produced minor effects on virulence in a mouse lung infection model, but ExoY did not play a major role in *P. aeruginosa* pathogenesis [44]. Aiello *et al.* developed two cellular reporter assays and screened a library of 80,000 compounds to search for inhibitors of *P. aeruginosa* type III secretion [45]. The primary assay consisted of a transcriptional fusion of the *Photorhabdus luminescens luxCDABE* operon to the *P. aeruginosa exoT* effector gene, and the secondary assay included direct measurements of T3SS-mediated secretion of a *P. aeruginosa* ExoS effector-β-lactamase fusion protein, as well as detecting the secretion of native ExoS. Five compounds (Figure 5) were identified and demonstrated inhibitory activity against *P. aeruginosa* type III secretion without affecting bacterial growth. These inhibitors also blocked the T3SS-mediated secretion of a YopE effector-β-lactamase fusion protein from an attenuated *Y. pestis* strain. The best inhibitor identified is phenoxyacetamide MBX1641 (**9**), which effectively inhibits the secretion of T3SS effector protein ExoS in *P. aeruginosa*. 

**Table 1:** Target specificity and *in vitro* antibacterial activity of three lead *N*-hydroxybenzimidazoles.

Compound	IC_50_ (μM) ^a^		
	LcrF	ExsA ^b^	SlyA ^c^
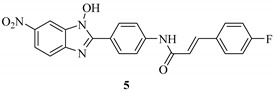	3.9	3.5	>53.8
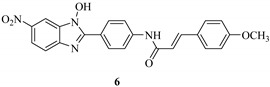	8.0	5.9	>55.2
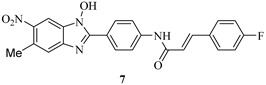	7.6	6.7	>52

^a^ IC_50_ was determined using a dose-response analysis with the maximum concentration of 25 μg/mL; ^b^ MAR transcription factor in *P. aeruginosa*; ^c^ MarR family transcription factor in *Salmonella* spp.

**Figure 6:**
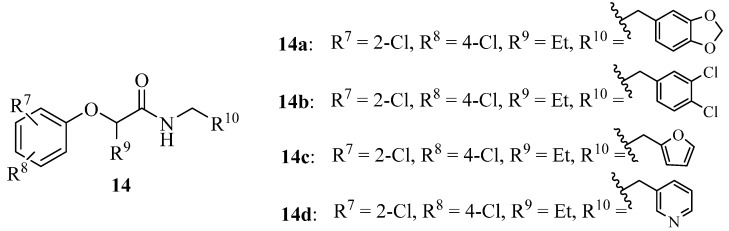
Structures of Compounds **14a**–**14d**.

**Figure 7:**
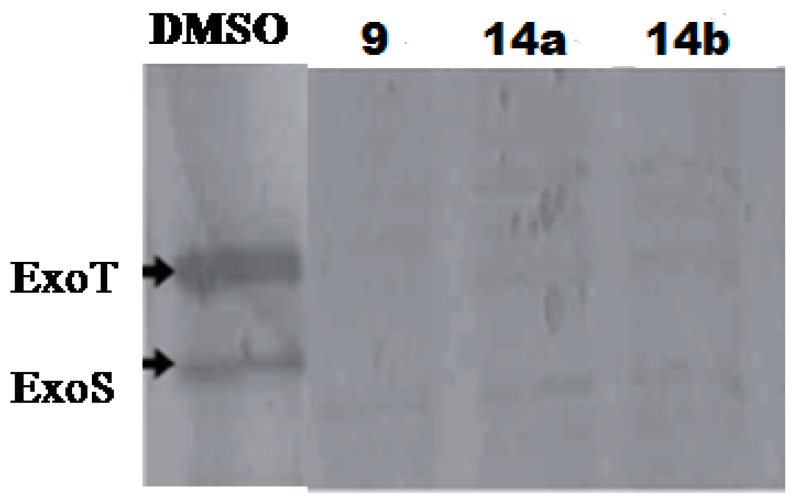
Alteration of the production of T3SS effectors ExoS and ExoT by T3SS inhibitors. *P. aeruginosa* PAO1 cells were grown in LB broth supplemented with 10 mM NTA (nitrilotriacetic acid) and 250 μM inhibitors. The same volume of DMSO was added to the culture as a negative control. The Western blot was performed using an anti-ExoS antibody.

In order to search for more potent T3SS inhibitors against *P. aeruginosa*, our lab studied the structure-activity relationship (SAR) of MBX1641 in collaboration with Yang’s group at the University of Wisconsin at Milwaukee. We designed and synthesized a series of new α-phenoxyacetamide derivatives by modifying the 2,4-dichlorophenoxy group, the length of the amide side chain and the 3,4-(methylenedioxy)benzyl group [46]. *P. aeruginosa* PAO1 was chosen as the target strain, and a promoter-probe vector pPROBE-AT containing the promoterless green fluorescent protein (*gfp*) gene was used to construct an *exoS* promoter-*gfp* plasmid reporter pATexoS. PAO1 containing pATexoS was cultured in T3SS-inducing medium containing DMSO or supplemented with 250 μM of each synthesized compound, and promoter activity was assessed at 6 h by measuring GFP intensity using flow cytometry. Four new derivatives (**14a**–**14d**; Figure 6) have shown a strong inhibitory effect against *exoS* gene expression in *P. aeruginosa.* Among them, **14d** not only exhibits stronger potency against PAO1 T3SS than MBX1641, but also has better solubility in aqueous solution. In addition, we selected a number of compounds to determine the expression level of ExoS and ExoT proteins via Western hybridization. MBX1641, **14a** and **14b** significantly inhibited the expression of ExoS and ExoT proteins (Figure 7). The results have demonstrated that the inhibitors can effectively suppress the expression of PAO1 T3SS effectors ExoS and ExoT. 

### 4.4. 2-Imino-5-arylidenethiazolidinones as T3SS Inhibitors

**Figure 8:**
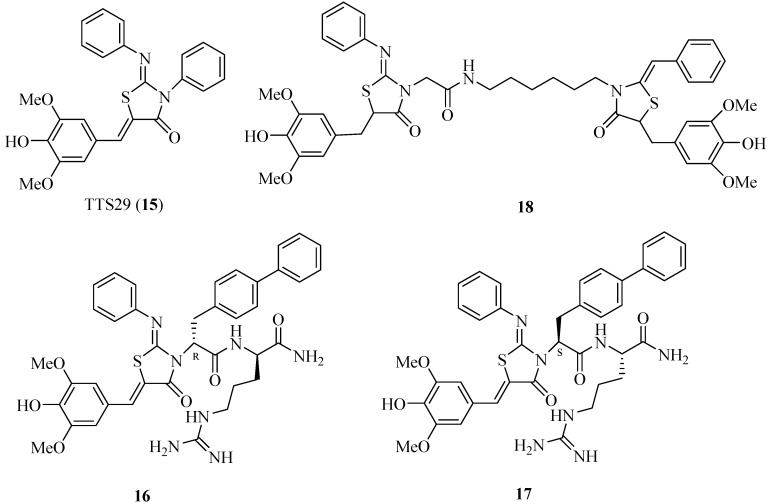
Structures of Compounds **15**–**18**.

In search of *S. typhimurium* T3SS inhibitors, Felise *et al*. developed and performed a high-throughput screening for compound libraries of 92,000 small molecules and discovered that 2-imino-5-arylidene thiazolidinone (TTS29; Figure 8) inhibited T3SS secretion or assembly without affecting bacterial growth [47]. They purified the needle complexes of *S. typhimurium* and found that TTS29-treated samples had lower levels of needle components, whereas the levels of whole-cell proteins were unchanged. These results indicated that TTS29 disrupts the needle assembly. This inhibitor can block the secretion and virulence functions of a wide array of animal and plant Gram-negative bacterial pathogens, such as *Y.* spp., *P. aeruginosa* and *Francisella novicida.* However, the major disadvantage for TTS29 is its moderate potency (only 83 μM). Therefore, the researchers synthesized a series of new derivatives of TTS29, and the structure-activity relationship results showed that imino nitrogen, the aryl group and the substitution pattern on the arylidene ring are critical for inhibitory activity, whereas the amido nitrogen is tolerated for modification. Dipeptide analogs **16** and **17** (Figure 8) have much better potency than TTS29 at low micromolar IC_50_ values (8 μM and 3 μM, respectively) [48]. The new analogs were synthesized through a solid-phase approach. Some tethered thiazolidinone dimers had also been synthesized, and many of these dimers inhibited the T3SS-dependent secretion of a virulence protein at a concentrations lower than that of TTS29 [49]. For example, the IC_50_ value of dimer **18** is 5 μM.

### 4.5. Plant Phenolic Compounds as T3SS Inhibitors

**Figure 9:**
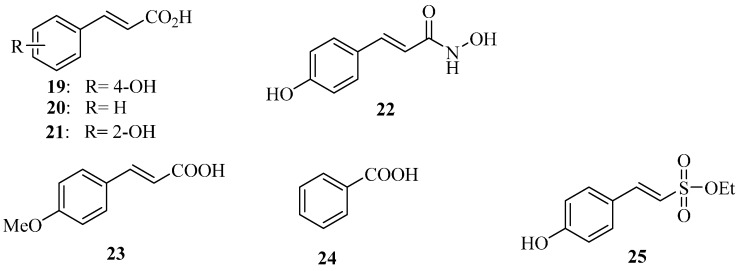
Structures of Compounds **19**–**25**.

Natural plant phenolic compounds are secondary metabolites synthesized by plants. Apart from their role in growth, pigmentation and reproduction, they also play an important role in disease resistance. Plants have evolved a systemic acquired resistance mechanism to protect themselves from pathogen invasion [50]. Inspired by this self-protection mechanism found in nature, we explored inhibitors of *D. dadantii* T3SS hrpA genes. After systematically screening a series of natural plant phenolic compounds and their derivatives, we found that *p*-coumaric acid (**19**) (Figure 9) significantly repressed the expression of T3SS regulatory genes through the *HrpS-Hrp* two-component system [51]. On the other hand, *trans*-cinnamic acid (**20**) and *o*-coumaric acid (**21**) induced the expression of *D. dadantii* T3SS genes *hrpA* via the RsmB-RsmA pathway. 

Very recently, we converted **19** into the corresponding hydroxamic acid and found that the resulting *trans*-4-hydroxycinnamohydroxamic acid (**22**) has an eight-fold higher inhibitory potency than **19** [52]. **22** inhibits the T3SS of *D. dadantii* HrpY via HrpX/HrpY two-component signal transduction and Rsm systems. To the best of our knowledge, this is the first inhibitor to affect the T3SS of *D. dadantii* through both the transcriptional and post-transcriptional pathways. In addition, our previous studies show that **22** reduces the biofilm formation of *P. aeruginosa* PAO1 and affects the T3SS of *P. aeruginosa* via the GacSA-RsmYZ-RsmA-ExsA regulatory pathway [53].

*E. amylovora*, of the family *Enterobacteriaceae*, is a Gram-negative bacterial plant pathogen. It is the causal agent of fire blight in apple, pear, raspberry and other rosaceous plant species, and it infects leaves, blossoms, succulent shoots and immature fruits. Annual losses to fire blight and costs of control in the USA are estimated at over $100 million. Losses and costs in many other countries are also huge [54]. Currently, the approach for controlling fire blight pathogen employs antibiotics and copper spray. The most commonly-used antibiotics for treating *E. amylovora* are streptomycin and oxytetracycline: streptomycin kills *E. amylovora*, whereas oxytetracycline merely inhibits its growth. In the 1990s, 30%–40% of planted pear trees in the USA were treated with streptomycin or oxytetracycline or both, and 19% of planted apple trees were treated with streptomycin each year [55]. However, the streptomycin-resistant strains of *E. amylovora* are now widespread and common in apple and pear orchards of the western United States and British Columbia in Canada and have also been reported in New Zealand, Israel and Lebanon. Resistance in the fire blight pathogen has had widespread economic and political implications [55]. The U.S. EPA strictly regulates the use of existing antibiotics (e.g., streptomycin and oxytetracycline) on plants in the USA and has established a higher standard for approval of new antibiotics due to antibiotic resistance concerns. 

We have found that some plant phenolic compounds can inhibit the expression of *E. amylovora* T3SS [56]*.* Using a green fluorescent protein (GFP) reporter combined with a high-throughput flow cytometry assay for measuring the expression of *E. amylovora* T3SS, 4-methoxycinnamic acid (**23**) and benzoic acid (**24**) (Figure 9) were identified as *E. amylovora* T3SS inhibitors. **23** and **24** altered the expression of *E. amylovora* T3SS via the *HrpS-HrpL* pathway. **23** inhibited the T3SS gene through the *RsmB-RsmA* system. We observed that the two inhibitors weakened the hypersensitive response (HR) in tobacco leaves by suppressing the T3SS of *E. amylovora*. Additionally, the sulfonate ester **25** was identified as the *E. amylovora* T3SS inducer [56]. The phenolic compounds (**23**, **24**) and their derivatives, which specifically targeted the *E. amylovora* T3SS, may provide an alternative strategy to antibiotics in fire blight control. 

### 4.6. Polyol Products as T3SS Inhibitors

**Figure 10:**
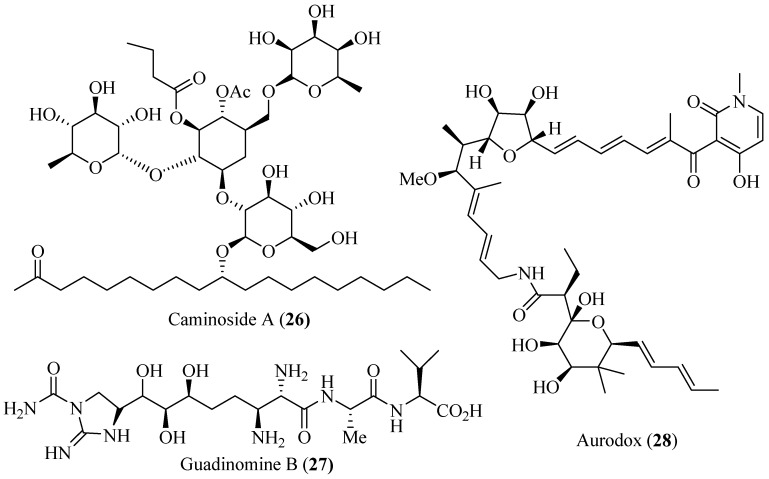
Structures of caminoside A, guadinomine B and aurodox.

Although the majority of *Escherichia coli* strains are benign for humans, several strains are pathogenic. For example, enteropathogenic *E. coli* (EPEC) infects the human intestinal epithelium and is a major cause of infantile diarrhea in developing countries. EPEC is an extracellular bacterial pathogen and uses a T3SS to deliver bacterial effectors into host cells [57]. Linington *et al.* isolated a complex glycolipid, caminoside A (**26**; Figure 10), from the marine sponge *Caminus sphaeroconia* [58]. Caminoside A effectively blocked the pathogenicity of EPEC by inhibiting the T3SS expression with an IC_50_ value of 20 μM and without killing the bacteria. Caminosides B, C and D were isolated from the marine sponge *C. sphaeroconia*, and all of the glycolipids showed inhibitory activity against the EPEC T3SS (IC_50_ = 20 μM) [59].

Six guadinomines were isolated from the culture broth of *Streptomyces* sp. K01-0509 as inhibitors of EPEC T3SS-induced hemolysis by Iwatsuki *et al.* [60,61]. Guadinomine B (**27**; Figure 10) showed the most potent inhibition with an IC_50_ value of 7 ng/mL. 

A linear polyketide, aurodox (**28**; Figure 10), was isolated from the culture broth of *S.* sp. by using a screening system for the T3SS-mediated hemolysis of EPEC [62]. Aurodox strongly inhibited the T3SS-mediated hemolysis with an IC_50_ value of 1.5 μg/mL without affecting bacterial growth, and it specifically blocked the secretion of T3SS proteins, such as EspB, EspF and Map. In an *in vivo* infection study, after aurodox was administered, mice survived a lethal dose of *Citrobacter rodentium*, a model bacterium for human pathogens, such as EPEC. This study suggests that certain microbes might produce small molecules capable of counteracting bacterial infection [62].

## 5. Conclusions and Prospects

In the past few years, extraordinary efforts have been made by a number of laboratories to discover new T3SS inhibitors as potential anti-infective agents. However, some challenges still need to be addressed before these inhibitors are proven to be effective therapeutics. None of the small-molecule T3SS inhibitors described in this review have advanced into the clinic. One major issue is that the precise mechanisms of action and effective targets remain unclear. Another is that the current small-molecule T3SS inhibitors have limited potency, with IC_50_ values in the single digits of micromolar concentrations.

Clinical development of the antibody KB001-A against *P. aeruginosa* infection has clearly demonstrated the therapeutic potential of T3SS inhibitors. Since the atomic structure of the needle body of *S. typhimurium* has been successfully revealed, it is hoped that the next major breakthroughs will be the complete structural elucidation of all of the T3SS structural proteins, effector proteins and chaperones. If this can be achieved, then clear targets for T3SS inhibitors will be within reach. The design and synthesis of small-molecule T3SS inhibitors will be more specific and potent, and researchers will more easily discover and develop such inhibitors for treating human bacterial infections.
